# Immunogenicity and therapeutic effects of a *Mycobacterium tuberculosis rv2190c* DNA vaccine in mice

**DOI:** 10.1186/s12865-017-0196-x

**Published:** 2017-02-27

**Authors:** Yan Liang, Xiaoyan Zhang, Xuejuan Bai, Li Xiao, Xiaomei Wang, Junxian Zhang, Yourong Yang, Jinying Song, Lan Wang, Xueqiong Wu

**Affiliations:** 1 Army Tuberculosis Prevention and Control Key Laboratory, Beijing Key Laboratory of New Techniques of Tuberculosis Diagnosis and Treatment, Institute of Tuberculosis Research, the 309th Hospital of Chinese PLA, Beijing, 100091 People’s Republic of China; 2 Zhengzhou Kingmed Center for Clinical Laboratory, Zhengzhou, 450016 People’s Republic of China

**Keywords:** DNA vaccine, *rv2190c* DNA, Immunotherapy, *Mycobacterium tuberculosis*

## Abstract

**Background:**

Tuberculosis (TB) is a major global public health problem. New treatment methods on TB are urgently demanded. In this study, *Mycobacterium tuberculosis* (MTB) *rv2190c* DNA vaccine was prepared and its immunogenicity and therapeutic effects were evaluated.

**Results:**

Non-infected mice immunized with *rv2190c* DNA or *ag85a* DNA showed higher levels of interferon-gamma (IFN-γ) in stimulated spleen lymphocyte culture supernatants, and had more Th1 cells and an elevatory ratio of Th1/Th2 immune cells in whole blood, indicating that Th1-type immune response was predominant. Compared with the saline group, *ag85a* DNA group and *rv2190c* DNA group in the infected mice decreased the lung colony-forming units (CFUs) by 0.533 and 0.283 log_10_, and spleen CFUs by 0.425 and 0.321 log_10_ respectively, and pathological lesion.

**Conclusions:**

The *rv2190c* DNA had some immunotherapeutic effect on TB.

**Electronic supplementary material:**

The online version of this article (doi:10.1186/s12865-017-0196-x) contains supplementary material, which is available to authorized users.

## Background

Tuberculosis (TB) is a severe respiratory infectious disease in which protective immunity and pathological hypersensitivity to an intracellular bacterium coexist [1, 2]. In 2013, there were 9 million incident cases and 1.5 million deaths [3]. It is difficult to cure with anti-TB chemotherapy because a long treatment duration (a minimum of six-month chemotherapy) with multiple drugs was used on active TB, many TB patients cannot complete a full period of treatment, which lead to failure of treatment and acquired drug resistance with eventual creation of multi-drug-resistant TB (MDR-TB) [4]. Accordingly, effective vaccines are needed in the face of failing drug treatments, and this may include therapeutic vaccines. Protective immunity against TB is largely attributed to a cellular immune response in which the production of the Th1-type cytokines (for example interferon-gamma, IFN-γ) predominates over the production of the Th2-type cytokines (for example interleukin-4, IL-4) [5–7]. DNA vaccination has been found to establish and boost antigen-specific cellular immunity in the direction of responses. Furthermore, immunotherapy with plasmid DNA has been found to be an effective adjunctive treatment in combination with antibacterial chemotherapy in mice. It both shortened the period of treatment and improved the therapy outcome of latent TB infection [8, 9]. Furthermore, we and others have shown that *ag85a* DNA vaccine can also have immunotherapeutic effects against MDR-TB in mice [10–12]. Here we report that the *rv2190c* gene of *Mycobacterium tuberculosis* (MTB) is similarly effective as a therapeutic DNA vaccine. The open reading frame (ORF) contains 1177 nucleotides and encodes a hypothetical protein with an NlpC/P60 domain [13]. Målen et al. [14] identified the protein product of *r*v2190c in MTB culture filtrate, showing that it could be expressed and secreted in vitro. Parthasarathy et al. [15] showed that the gene and its protein product were essential for normal growth and virulence of MTB in vivo and McMurry et al. [16] found that Rv2190c peptide could stimulate peripheral blood mononuclear cells to secrete IFN-γ in persons with latent TB infection. Beyond that, MTB Rv2190c antigen has not been extensively studied and thus therefore presents new possibilities for developing drug targets, diagnostic reagents and vaccines. Accordingly we evaluated its immunogenicity and immunotherapeutic effects as a DNA vaccine in mice.

## Methods

### Mice

One hundred 6-8 week age of female BALB/c mice without specific pathogen were purchased from the Academy of Military Medicine and Science, Beijing, China, maintained under infection barrier conditions in a negative pressure animal room in the 309^th^ Hospital of Chinese PLA, Beijing, China, and fed a sterile commercial mouse diet (Beijing KeAoXieLi Company Limited, China). The study procedures were approved by the 309th Hospital of the Chinese PLA Research Animal Ethics Committees.

### MTB strain

MTB H_37_Rv was provided by National Institutes for Food and Drug Control, Beijing, China.

### Preparation of recombinant Rv2190c protein

The procedures preparation of Rv2190c protein were briefly as follows: a 1168 bp gene fragment of *rv2190c* was amplified by polymerase chain reaction (PCR). The forward primer with a *Nhe I* enzyme site: 5′- CTA*GCTAGC*ATGAGGCTCGACCAGAGGTGGTT -3′; the reverse primer with an *EcoR I* enzyme site: 5′- CCG*GAATTC*TCAGTAACGGCGGGCGTCG -3′. The resulting PCR product was ligated with plasmid vector pET30a (Promega), and then transformed into *E. coli* DH5α [17]. The recombinant plasmid *rv2190c/pET30a* was analyzed by Huada gene Ltd. Beijing, China and had 100% identity with the designed sequence by BLAST analysis. The recombinant plasmid was transformed into *E. coli* strain BL21 (DE3). The expression and purification of recombinant Rv2190c protein was performed according to the method described previously [18].

### Construction of *rv2190c* DNA vaccine

**Fig. 1:**
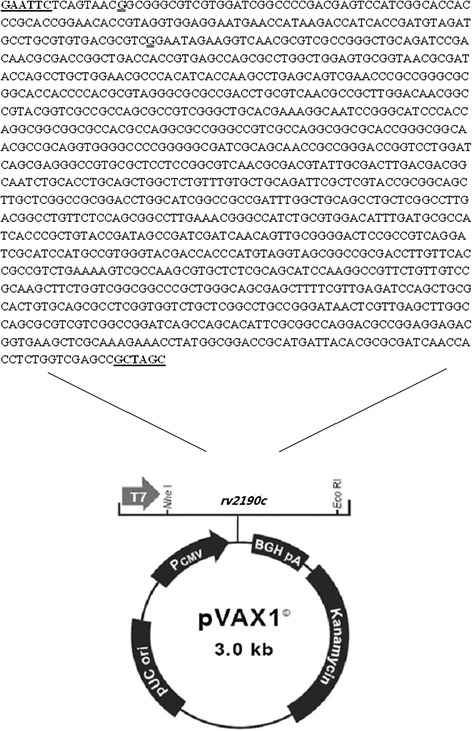
The construction map and gene sequence of *rv2190c* DNA. *Black* letters are the *rv2190c* gene sequence. The *bold* and *underline* letters are the site of restrictive enzymes *Nhe I* and *EcoR I*. The *double underline* letters are the optimized gene sequence

The construction map and method of *rv2190c* DNA vaccine were shown in Fig.1 and described previously [19]. The sequence encoding Rv2190c was amplified from MTB H_37_Rv by PCR. The forward primer with a *Nde I* enzyme site: 5′- CTAG*GCTAGC*CACCATGGGGCTCGACCAGAGGTGGTT-3′; the reverse primer with an *EcoR I* enzyme site: 5′-CCG*GAATTC*TCA GTAACGGCGGGCGTCG -3′ (synthesized by Shanghai Sangon Ltd. Beijing, China). The PCR product fragment was 1177 bp. Recombinant plasmid *rv2190c* DNA was sequenced by Huada gene Ltd. Beijing, China and was found to conform to the sequence designed using BLAST analysis. EndoFree plasmid purification kit (Qiagen, Hilden, Germany) was used to purify *rv2190c* DNA vaccine.

### Immunogenicity of *rv2190c* DNA vaccine

Fifty female BALB/c mice were divided into 5 groups as follow: (1) saline as a negative control (100 μl); (2) vector pVAX1 as a negative control (100 μg in 100 μl saline); (3) *M. vaccae* (22.5 μg in 100 μl saline, Longcome Biological Pharmacy, Anhui, China) as a positive control; (4) *ag85a* DNA (100 μg in 100 μl saline), as a positive control; (5) *rv2190c* DNA (100 μg in 100 μl saline), immunized intramuscularly three times at two-week intervals.

### Cytokine production in vitro

The mice were sacrificed at three weeks after the third immunization. The mouse splenocytes were isolated and cultured (5 × 10^5^ cells/well) with Ag85A or Rv2190c protein (20 μg/ml) or phytohaemagglutinin (PHA; 20 μg/ml) for 72 h. The levels of IFN-γ and IL-4 in the splenocytes culture supernatants were detected using enzyme-linked immunosorbent assay (ELISA) kit (BD PharMingen, San Diego, California, USA) according to the manufacturer’s procedures.

### Determination of CD4^+^ T cell subsets expressing intracellular IFN-γ or IL-4

The operation procedure was described previously [20]. Briefly, Th1 and Th2 cells responding to Ag85A or Rv2190c proteins were calculated. Cells expressing IFN-γ and IL-4 were presented as a percentage of the total population of CD3^+^ cells. The data were collected using a fluorescence-activated-cell-sorting (FACS) Calibur flow cytometer (BD Pharmingen) and analyzed using CellQuest software.

### Treatment of TB-infected mice

Fifty mice were challenged with 6.4 × 10^5^ colony-forming units (CFUs) of MTB H_37_Rv through trail vein injection*,* randomly divided into five groups mentioned above, and then treated at the third day after infection.

### Bacterium counts

The mice were sacrificed by cervical dislocation under anesthetic with 5 ml dethyl ether (Beijing Chemical Reagents Company, Beijing, China) at two weeks after the third immunization. The tissue suspensions of mouse lungs and spleens were serially diluted 10-fold, and 100 μL suspension dilution were inoculated in duplicate on Lowenstein-Jensen medium plates and cultured at 37 °C for 4 weeks. MTB colonies on medium were counted and the results were showed as CFUs per organ.

### Lung histopathological examination

The mouse lungs tissues paraffin-embedded were sliced into 3-μm thick tissue sections, which were dyed with hematoxylin and eosin, and then examined by a certified and veteran pathologist.

### Statistical analyses

Data are shown as means and standard deviations. Statistical analyses were performed using one-way ANOVA followed by Dunnett’s multiple comparison test, and a *P*-value of < 0.05 was considered as significant difference.

## Results

### Preparations of *rv2190c* DNA and recombinant Rv2190c protein

The nucleotide sequence in pVAX1-*rv2190c* plasmid had 100% identity with MTB *rv2190c* sequence as designed and the fragment size from restriction-enzyme-digested recombinant plasmid pVAX1-*rv2190c* was 1.177 kb on 0.8% agarose gel electrophoresis, and that confirmed the successful construction (Additional file 1: Figure S1).

The recombinant Rv2190c protein was soluble in expression and amounted to 30% of total bacterial protein. The molecular mass of the purified Rv2190c protein was around 42 kDa by SDS-PAGE, its purity was higher than 90% (Additional file 2: Figure S2).

### Specific cytokine production levels in splenic lymphocyte culture supernatants

**Fig. 2:**
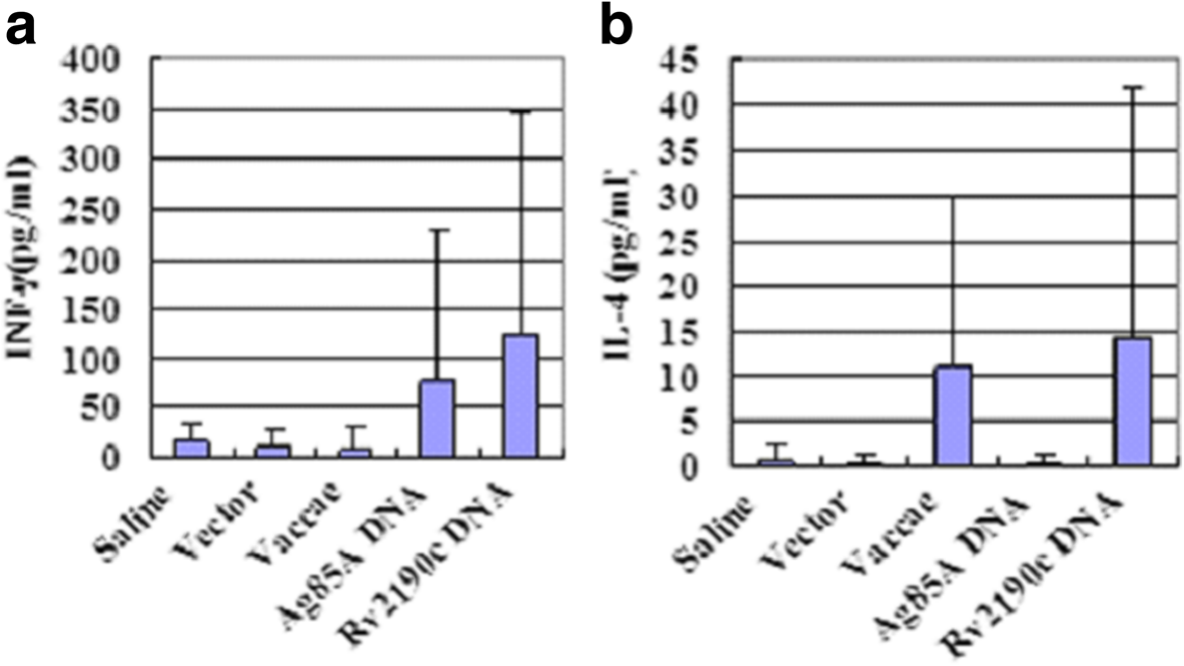
IFN-γ (**a**) and IL-4 (**b**) levels in the culture supernatants of splenocytes were detected by ELISA. The production of IFN-γ from *rv2190c* DNA group was significantly higher than from the saline group, vector group and *M. vaccae* group (*P* < 0.05), but the production of IL-4 was not significantly different between the groups

The IFN-γ level in splenic lymphocyte culture supernatants in the *rv2190c* DNA group was obviously higher than those in the saline, plasmid vector and *M. vaccae* groups (*P* < 0.05), but had no significant difference from that in the *ag85a* DNA group. The production of IL-4 was not significantly different between groups (Fig.2, Additional file 3).

### CD4^+^ T cell subsets expressing intracellular IFN-γ or IL-4

**Fig. 3:**
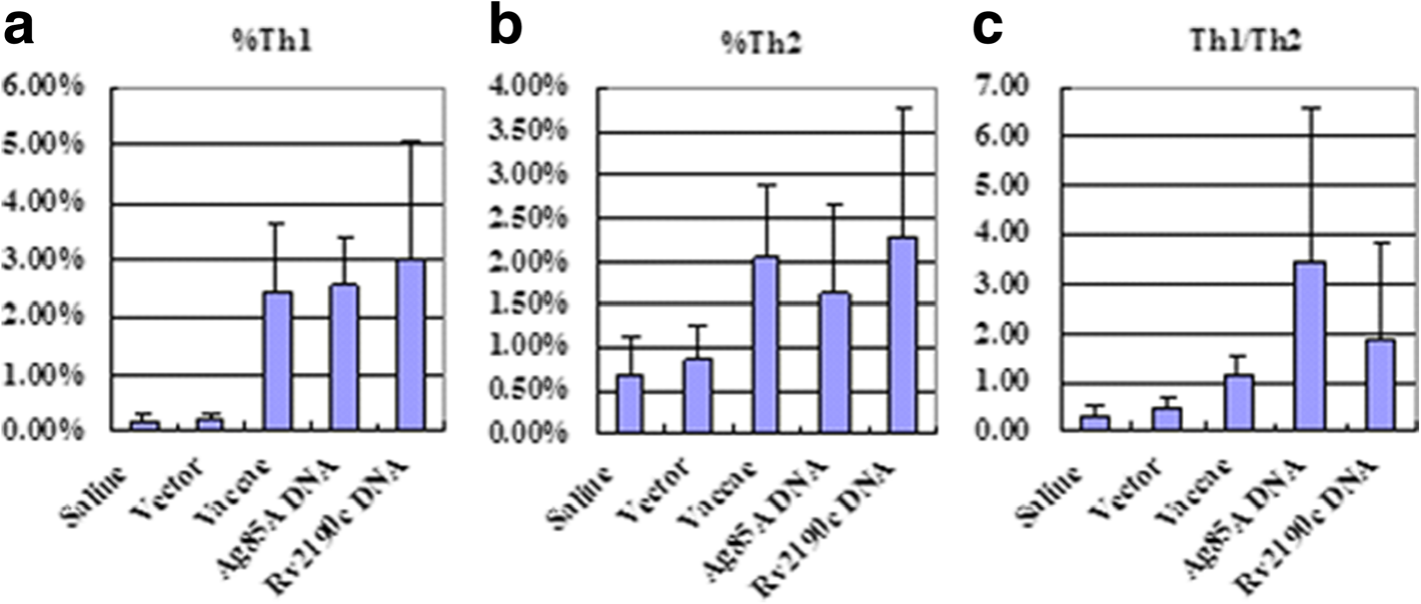
Frequencies of CD4+ T cell subsets in whole blood assessed by flow cytometry. The data are expressed as the mean % ± standard deviation. **a** Th1 cells expressing IFN-γ (IFN-γ-FITC); **b** Th2 cells expressing IL-4 (IL-4-PE); **c** The ratio of Th1/Th2 cell frequencies

The proportion of CD4+ T cells expressing IFN-γ (Th1) in response to Ag85A or Rv2190c proteins was significantly higher in the whole blood from the *rv2190c* DNA group than those from the saline group and vector group by flow cytometry (*P* < 0.001), but there was no significant difference from the *M. vaccae* group or *ag85a* DNA group. The proportion of cells expressing IL-4 (Th2) was significantly higher in the blood from the *rv2190c* DNA group than those from the control groups (*P* < 0.05), but there was no significant difference from the *M. vaccae* group or *ag85a* DNA group. Th1/Th2 ratio in the blood from the *rv2190c* DNA group was significantly decreased than that in the *ag85a* DNA group, and increased than those in the saline, vector and *M. vaccae* groups, but the differences were not significant (Fig. 3, Additional file 3).

### Mouse survival

There was one mouse death in vector group, one in *M. vaccae* group and one in *rv2190c* DNA group at 29 days after infection (90% survival). The other mice were all alive.

### Bacterial counts in the lungs and spleens

**Fig. 4:**
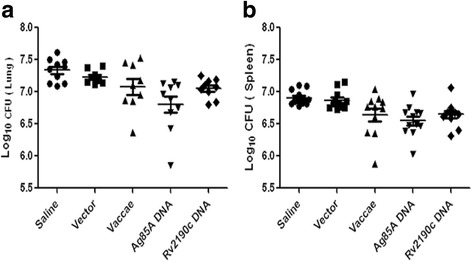
The numbers of live bacteria in lungs (**a**) and spleens (**b**) at six weeks after infection. Compared with the saline group, *rv2190c* DNA and *ag85a* DNA reduced the pulmonary bacterial loads by 0.283 (*P* > 0.05) and 0.533 logs (*P* < 0.05) and the splenic bacterial loads by 0.321 (*P* < 0.05) and 0.425 logs (*P* < 0.05), respectively

The live bacteria in mouse lungs and spleens were determined at two weeks after third of immunotherapy. The lung CFUs from saline, vector, *M. vaccae*, *ag85a* DNA and *rv2190c* DNA groups were 7.334 ± 0.180, 7.233 ± 0.102, 7.081 ± 0.369, 6.801 ± 0.407 and 7.051 ± 0.154 log_10_, respectively (Fig.4a, Additional file 3), and the spleen CFUs were 6.919 ± 0.117, 6.808 ± 0.067, 6.652 ± 0.345, 6.494 ± 0.211 and 6.598 ± 0.143 log_10,_ respectively (Fig.4b, Additional file 3). Compared with saline group, *rv2190c* DNA and *ag85a* DNA decreased the lung CFUs by 0.283 (*P* > 0.05) and 0.533 log_10_ (*P* < 0.05) and the spleen CFUs by 0.321 and 0.425 log_10_ (*P* < 0.05), respectively.

### Histopathological changes

**Fig. 5:**
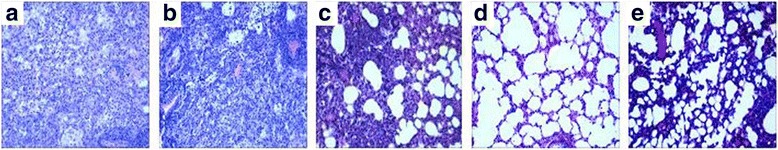
Pulmonary histopathological changes. This figure shows representative photomicrographs (H&E, 100×) of lung tissue obtained from mice each group at six weeks after infection with MTB H37Rv. **a** Saline group. **b** Vector group. **c** *M. vaccae* group. **d** *ag85a* DNA group. **e** *rv2190c* DNA group

The lung sections from the saline group and plasmid vector group showed extensive lung lesions, in which hyperemia and congestion in alveoli with many lymphocytes and destructive structure caused by severe inflammation. The lung sections from treatment groups showed more foamy macrophages but less lymphocyte infiltration, the alveoli were relatively clear and had normal structure. Representative histopathological changes of five groups were shown in Fig.5.

## Discussion

In this study, a DNA sequence encoding MTB Rv2190c protein was inserted into plasmid vectors pET30a and pVAX1, thereby recombinant Rv2190c protein and *rv2190c* DNA vaccine were prepared and subsequently used to compare the therapeutic effects of *rv2190c* DNA with those of *ag85a* DNA as vaccines against TB in mice.

The cellular immune responses to *rv2190c* DNA were measured as increase in frequency of circulating T cells that produced either IFN-γ or IL-4 in response to cognate vaccine antigen since these cytokines are markers of protective Th1 responses and non-protective Th2 responses respectively. A balance between Th1 and Th2 in which Th1 predominates is essential for the control of TB and mycobacterial infection in mice and human [2, 4]. In this study, the significantly increased IFN-γ level in the spleen lymphocyte culture supernatant, the abundance of Th1 cells and elevatory ratios of Th1 /Th2 cells in antigen-specifically stimulated whole blood from the *rv2190c* DNA group were all similar to the results obtained in the *ag85a* DNA group. Thus both vaccines induced a predominantly Th1 immune response [5, 12–14]. This finding was consistent with evidence from McMurry et al. [16] showing that Rv2190c peptide can induce IFN-γ production in cells from persons with latent TB infection, and indicated a potential utility of Rv2190c antigen in therapeutic vaccination.

When used to treat infected mice, *rv2190c* DNA vaccine did indeed reduce the numbers of live bacteria found in the organs sampled after 6 weeks of treatment when compared to control mice. However, the reduction in lung bacterial load was small, not statistically significant, and less than that obtained after *ag85a* DNA treatment. In contrast, the reduction of load in the spleen was significant, but again less than that observed after *ag85a* DNA treatment. Apparently, although the rv2190c protein enhanced antibacterial immunity it was in this respect a less effective vaccine antigen than the Ag85A protein. Nevertheless, in a practical therapeutic vaccine for human use, more than one antigen is likely to be required and Rv2190c is clearly a candidate for inclusion. The greater effects in spleen than in lung suggest that the DNA vaccines were more active against bacteria in extra-pulmonary sites than in the lung, but whether this was due to differential effects on bacterial dissemination, growth inhibition or killing was not investigated. Although organ bacterial load is one of the important indicators to evaluate curative effects on animal TB experiments [21], evaluation of the impact upon pathology is also essential. Strikingly, the lesions in the lungs of *rv2190c* DNA and *ag85a* DNA vaccinated groups were similarly lessened, suggesting that *rv2190c* DNA and *ag85a* DNA provided similarly efficient immunotherapy for TB disease in this model.

## Conclusions

We successfully constructed a MTB *rv2190c* DNA vaccine that could induce Th1-type cellular immune reactions in mice and had some immunotherapeutic effects on tuberculosis in mice. It may have potential for use in an immunotherapeutic DNA vaccine against TB.

## Additional files

Additional file 1: Figure S1. Fragment sizes of restriction-enzyme-digested recombinant plasmid *pVAX1-rv2190c*. 1. PCR amplification product of the recombinant plasmid *pVAX1-rv2190c* colony; 2. The recombinant plasmid *pVAX1-rv2190c* digested by restriction endonuclease *Nhe I* and *EcoR I*; 3. Digest of the pVAX1 vector DNA with restriction endonuclease *Nhe I* and *EcoR I*; 4. The recombinant plasmid *pVAX1-rv2190c* DNA digested by restriction endonuclease *EcoR I*; 5. Digest of the pVAX1 Vector DNA with restriction endonuclease *EcoR I*; M: DM5000 DNA Marker. (TIF 45 kb)
Additional file 2: Figure S2. Expression and purification of recombinant Rv2190c protein as determined by SDS-PAGE electrophoresis. The gel was subjected to electrophoresis followed by Coomassie blue staining. Lane M, protein molecular weight marker. Lane 1, *E. coli* lysates engineered before isopropylthiogalactopyranoside (IPTG) induction. Lane 2, *E. coli* lysates engineered after induction with IPTG. Lane 3, the purified recombinant Rv2190c protein. (TIF 39 kb)
Additional file 3: The Excel data file [FOLT] Figshare, [DOI:10.6084/m9.figshare.4668148 and https://figshare.com/s/bd46c22986c673579bb6] includes all datasets supporting the conclusions of this article: IFN-γ in spleen lymphocyte culture supernatants, IL-4 in spleen lymphocyte culture supernatants, CD4+ T cell subsets expressing intracellular IFN-γ or IL-4, CFU in the lungs and spleens.. (XLS 143 kb)

## Abbreviations

CFUs: Colony-forming units
ELISA: Enzyme-linked immunosorbent assay
FACS: Fluorescence-activated-cell-sorting
IFN-γ: Interferon-gamma
IL-4: Interleukin-4
MDR-TB: Multi-drug-resistant tuberculosis
MTB: *Mycobacterium tuberculosis*
ORF: Open reading frame
PCR: Polymerase chain reaction
TB: Tuberculosis
