# Supplementary figures and images for: Immunogenicity and therapeutic effects of a Mycobacterium tuberculosis rv2190c DNA vaccine in mice
Source: BMC Immunol. 2017 Feb 27;18:11. doi: 10.1186/s12865-017-0196-x (PMC5327546; doi:10.1186/s12865-017-0196-x)

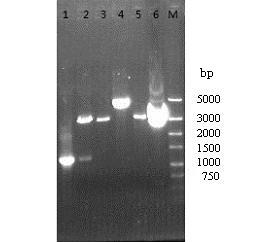

Supplement: Additional file 1: Figure S1. — Fragment sizes of restriction-enzyme-digested recombinant plasmid pVAX1-rv2190c. 1. PCR amplification product of the recombinant plasmid pVAX1-rv2190c colony; 2. The recombinant plasmid pVAX1-rv2190c digested by restriction endonuclease Nhe I and EcoR I; 3. Digest of the pVAX1 vector DNA with restriction endonuclease Nhe I and EcoR I; 4. The recombinant plasmid pVAX1-rv2190c DNA digested by restriction endonuclease EcoR I; 5. Digest of the pVAX1 Vector DNA with restriction endonuclease EcoR I; M: DM5000 DNA Marker. (TIF 45 kb) [file 12865_2017_196_MOESM1_ESM.tif]

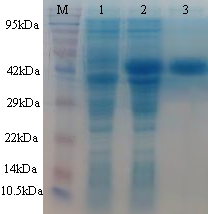

Supplement: Additional file 2: Figure S2. — Expression and purification of recombinant Rv2190c protein as determined by SDS-PAGE electrophoresis. The gel was subjected to electrophoresis followed by Coomassie blue staining. Lane M, protein molecular weight marker. Lane 1, E. coli lysates engineered before isopropylthiogalactopyranoside (IPTG) induction. Lane 2, E. coli lysates engineered after induction with IPTG. Lane 3, the purified recombinant Rv2190c protein. (TIF 39 kb) [file 12865_2017_196_MOESM2_ESM.tif]
